# The complete mitochondrial genome of *Trictenotoma davidi* Deyrolle, 1875 (Coleoptera: Trictenotomidae)

**DOI:** 10.1080/23802359.2021.1942266

**Published:** 2021-06-21

**Authors:** Liangjing Sheng, Tong Zhou, Zengzeng Shi, Xufei Pan, Xiaoqian Weng, Jiayi Ma, Songqing Wu

**Affiliations:** a College of Forestry, Fujian Agriculture and Forestry University, Fuzhou, China; b Key Laboratory of Integrated Pest Management in Ecological Forests, Fujian Province University, Fujian Agriculture and Forestry University, Fuzhou, China; c College of Life Science, Fujian Agriculture and Forestry University, Fuzhou, China

**Keywords:** Complete mitochondrial genome, *Trictenotoma davidi* Deyrolle, phylogenetic analysis

## Abstract

*Trictenotoma davidi* Deyrolle, 1875 is a beetle of the Trictenotomidae family. The length of the complete mitochondria genome of *T. davidi* was 15,910 bp with 24.1% GC content, including 39.9% A, 15.1% C, 9.0% G, and 36.0% T. The genome encoded 13 protein-coding genes, 22 tRNAs, and 2 rRNAs. Phylogenetic analysis showed that *T. davidi* was closely related to *Vincenzellus ruficollis*. This study provided useful genetic information for the evolution of *T. davidi* and Trictenotomidae insects.

*Trictenotoma davidi* Deyrolle, 1875 belongs to Trictenotomidae, a small family of beetles in the suborder Polyphaga, including two genera and fifteen species. Adult Trictenotomidae can be mistaken for Cerambycidae (Prioninae) or Lucanidae, but their 5-5-4 tarsal formula makes them distinctive (Beutel and Friedrich 2005). Based on larval characters as sequence-based studies, the family is considered to be closely related to the Salpingidae (Hu et al. 2020). They breed in wood and the eggs being laid under the bark. The larvae build tunnels in softwood in which they stay. The larvae are carnivorous and can be cannibalistic. Adults feed on tree sap (Lin and Hu 2019). *Trictenotoma davidi* was found in China and Vietnam where they live in montane forest habitats. However, no reports about the genetic evolution analysis of *T. davidi* have been published until now. In this study, we reported the complete mitochondrial genome of *T. davidi* based on Illumina sequencing data and investigated the phylogenetic relationship by the maximum-likelihood tree inference method. The results could provide important genetic information to study the genetic evolution of *T. davidi*.

The *T. davidi* adults were collected from Minhou county, Fujian province, China (119°33′98′′E, 25°84′65′′N) by the traps with sexual attractants. The specimens were deposited at the Fujian Agriculture and Forestry University (URL: https://lxy.fafu.edu.cn, contact person: Jiayi Ma and email: 790167087@qq.com) under the voucher number TN-202007. Total genomic DNA was extracted from an adult using TruSeq DNA Sample Preparation Kit (Vazyme, China), and purified by QIAquick Gel Extraction Kit (Qiagen, GER). DNA quality and concentration were determined using Nanodrop (Thermo Fisher Scientific, USA). DNA sequencing was performed by Illumina Hiseq 2500 (Illumina, USA). A total of 56,445,298 clean reads were obtained from the 58,712,478 raw reads after filtration. The clean reads were assembled by using MitoZ and metaSPAdes (Nurk et al. 2017). Then the assembly sequence was annotated by the MITOs webserver (Matthias et al. 2013). And tRNA genes were predicted using tRNAscan software (Lowe and Eddy 1997). The complete mitochondrial genome sequence of *T. davidi* has been submitted to NCBI GenBank with accession number MW580860. The complete mitochondria genome of *T. davidi* forms a circular structure covering 15,910 bp in length, with 13 protein-coding genes, 22 tRNAs, and 2 rRNAs. The GC content of the complete genome was 24.1%, the content of each base was 39.9% A, 15.1% C, 9.0% G, and 36.0% T.

To confirm the phylogenetic position of *T. davidi*, the evolutionary tree was constructed with related 15 different Coleoptera insects, including three Tenebrionoidea insects, eleven Elateroidea insects, and a Chrysomeloidea insect which as an outgroup, by MEGAX using the Maximum Likelihood tree model with 1000 bootstrap replicates (Hall 2013). The phylogenetic tree showed that the *T. davidi* was in a close relationship with *Vincenzellus ruficollis* Panzer, 1794 (Coleoptera: Salpingidae) (Figure 1). The complete mitochondrial genome of *T. davidi* will provide useful genetic information for increasing the richness of the Trictenotomidae, as well as assisting in phylogenetic and evolutionary studies of Trictenotomidae.

**Figure 1.**
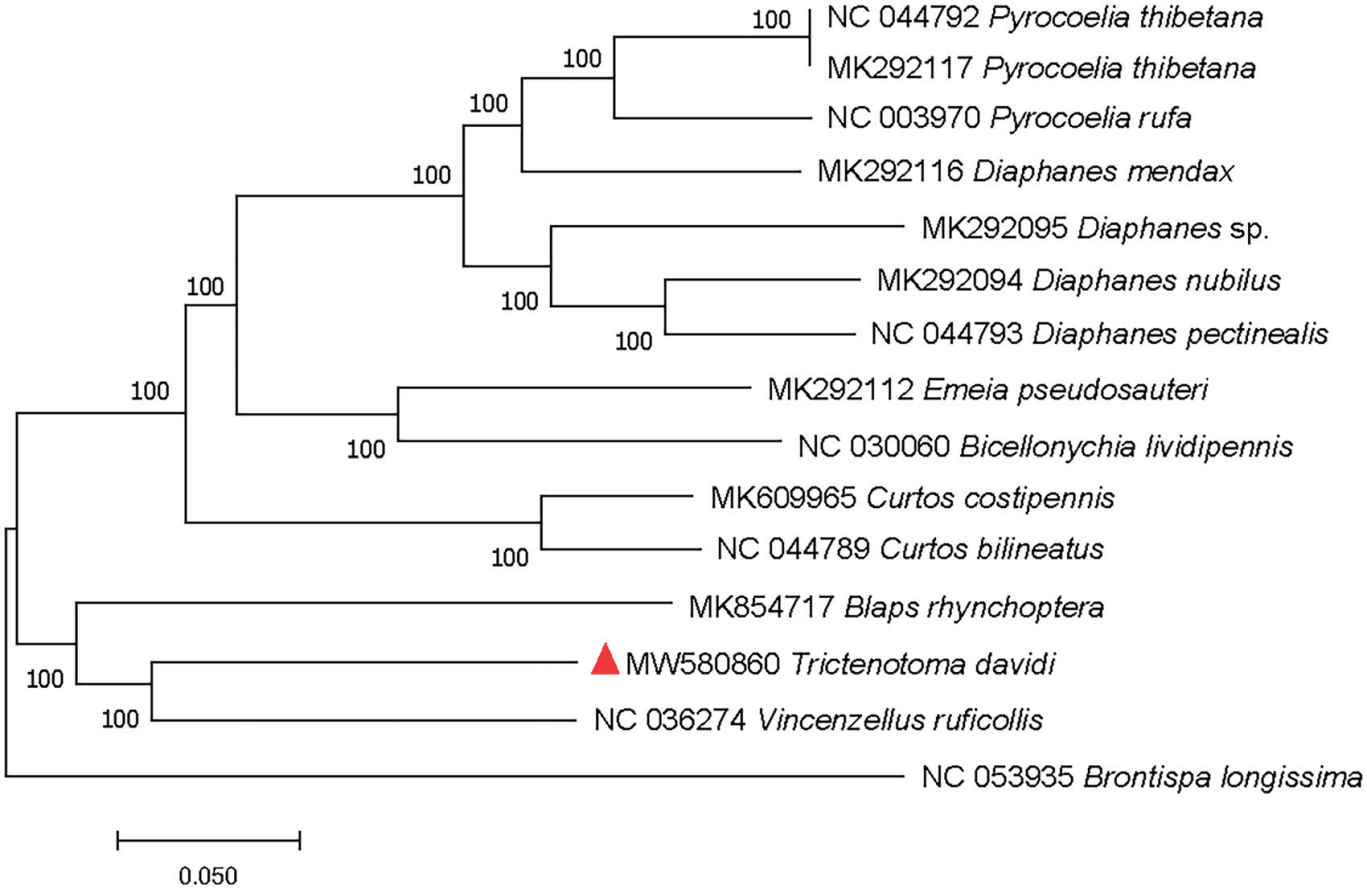
Maximum-likelihood tree of *Trictenotoma davidi* Deyrolle and related 14 different Coleoptera insects based on the mitochondrial genome. Bootstrap support values are labeled near the branch.

## Data Availability

The genome sequence data that support the findings of this study are openly available in GenBank of NCBI at https://www.ncbi.nlm.nih.gov under the assession no. MW580860. The associated BioProject, SRA, and Bio-Sample number was PRJNA702599, SRR13743949, and SAMN17975908, respectively.
